# Supplementary material for: Interactions of SARS-CoV-2, influenza and respiratory syncytial virus influence epidemic timing and risk
Source: Commun Med (Lond). 2026 Mar 14;6:259. doi: 10.1038/s43856-026-01504-x (PMC13133120; doi:10.1038/s43856-026-01504-x)
Supplement: Supplementary file 2 — Supplementary Information [file 43856_2026_1504_MOESM2_ESM.docx]

**Supplementary Information to:**

**Interactions of SARS-CoV-2, influenza and respiratory syncytial virus influence epidemic timing and risk**

**Contents:**

- Data sources for the positivity rates of viruses
- Supplementary Figures 1 to 13
- Supplementary Tables 1 to 19
- References

1. **Data sources for the positivity rates of SARS-CoV-2, IAV, IBV and RSV.**

The weekly pathogen positivity rates of respiratory viruses, SARS-CoV-2, IAV/IBV, and RSV, were collected for the United States (divided into 10 U.S. Department of Health and Human Services [HHS] regions), England (the United Kingdom), Denmark, Ireland, Portugal, Slovenia and Beijing from the following sources. We considered these countries based on the data availability and data quality. We collected global surveillance data on three types of viruses from the winter of 2021 to the spring of 2024. Only the United States, England (United Kingdom), Hong Kong (China), and countries within the European Union had publicly available weekly surveillance data for all four viruses. Hong Kong was excluded due to limited data. As COVID-19 control measures continued until the end of 2022, respiratory viruses remained at low prevalence during the 2021/2022 season and only returned to normal levels in 2023. The reporting quality varied across EU countries, to ensure data robustness, we retained only countries where weekly detection data available for more than 90% of the study period, with weekly sample sizes exceeding 200 per virus. Finally, we included only seven different locations and countries in the study.

**USA:** We collated the virus positivity rate data for 10 HHS regions in the United States, obtained from the National Respiratory and Enteric Virus Surveillance System (NREVSS)^1^, a sentinel network of laboratories located through the US. Data included the weekly number of specimens tested and the number testing positive for SARS-CoV-2, IAV, IBV and RSV, all samples were tested by reverse transcriptase-polymerase chain reaction (RT-PCR). The tested specimens are typically from patients who seek medical care due to respiratory symptoms.

**England:** We obtained the weekly positivity rates from the Respiratory DataMart System^2^, including the weekly number of specimens tested and the number testing positive for SARS-CoV-2, IAV, IBV and RSV. The Respiratory DataMart system is based on a sample of sentinel laboratories, testing for samples is performed using polymerase chain reaction (PCR) tests, most testing reported in Respiratory DataMart is done in patients with a clinical indication for a respiratory virus test.

**Ireland, Denmark, Portugal, and Slovenia:** We obtained the positivity rates for SARS-CoV-2, IAV, IBV and RSV from the European Surveillance System (TESSy)^3^, including the weekly number of specimens tested and the number testing positive for each virus, all samples for laboratory testing for selected respiratory viruses, regardless of whether the patient meets a syndromic case definition.

**Beijing:** We obtained the positivity rates for SARS-CoV-2 and IAV from the Virological Surveillance System in Beijing, which covers 39 sentinel hospitals across the city. Data included the weekly number of specimens tested and the number testing positive for SARS-CoV-2 and IAV, all samples was tested by real-time quantitative PCR, the tested population all consisted of influenza-like illness cases. Due to the small sample size for RSV pathogen detection in the historical data from Beijing, with approximately 100 people tested per week, the data exhibits significant fluctuations^4^ . Therefore, we did not include the RSV data from Beijing.

**Figure S1: Schematic diagram of two-pathogen interactive transmission model.**

{1,2} denotes the infection status of individuals with respect to virus 1 and virus 2. whereby S =susceptible, I = infectious refractory phase, P = noninfectious refractory phase, R = immunity phase, and subscripts 1 and 2 denote the corresponding virus-specific parameters.


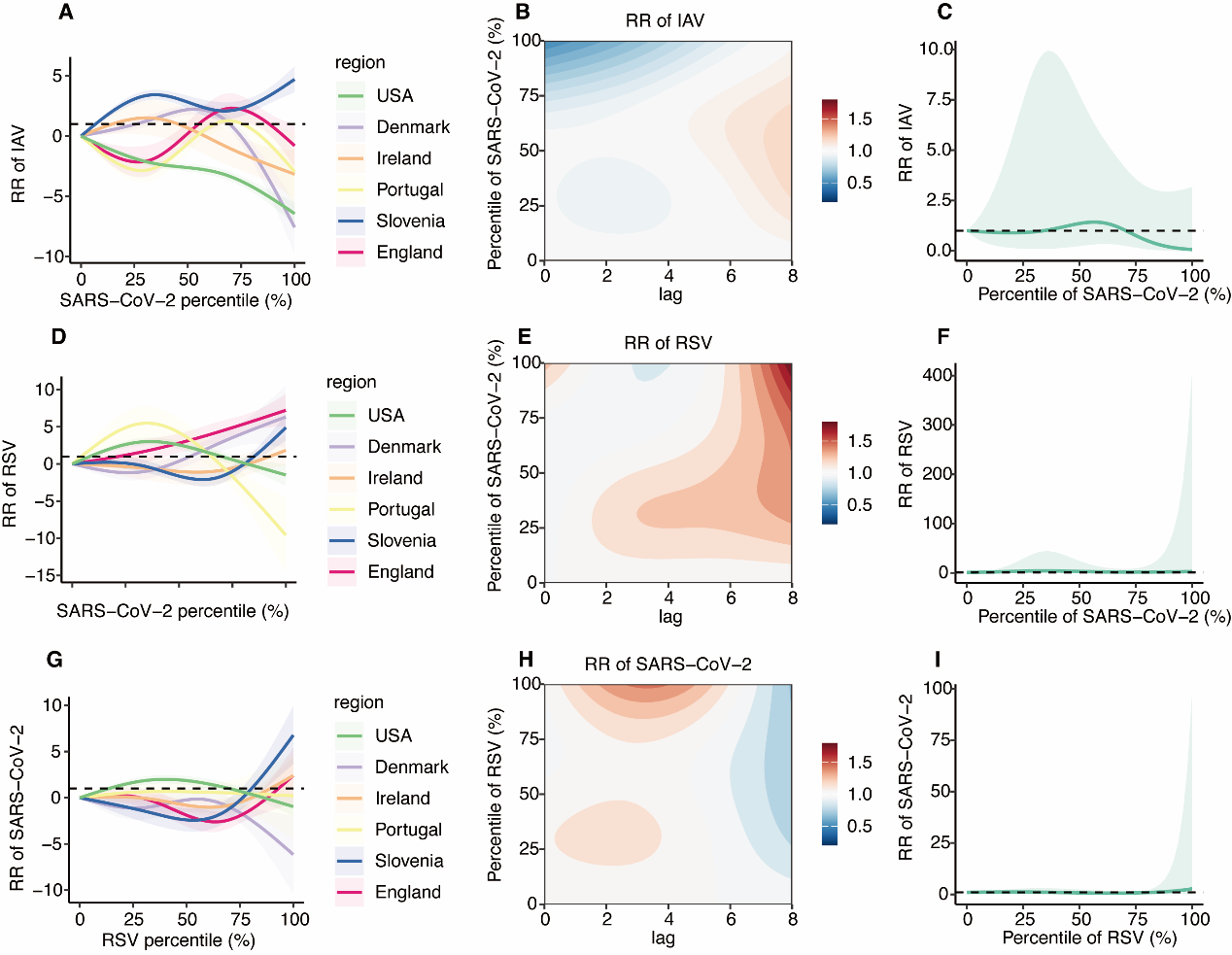


**Figure S2: Exposure-response association between viruses.** Associations between percentile of SARS-CoV-2 positivity and subsequent IAV infection risk. (A) RR of IAV associated with different percentiles of SARS-CoV-2 positivity across regions, shaded areas representing 95% CrI. (B) Pooled contour plot of the lag–response association between percentile of SARS-CoV-2 positivity and IAV risk, relative to the minimum SARS-CoV-2 percentile. (C) Pooled exposure–response association between percentile of SARS-CoV-2 positivity and IAV risk, shaded areas representing 95% CrI. (D–F) Associations between percentile of SARS-CoV-2 positivity and subsequent RSV infection risk. (G–I) Associations between percentile of RSV positivity and subsequent SARS-CoV-2 infection risk. Estimates are based on n = 6168 biologically independent weekly observations included in the DLNM.

**
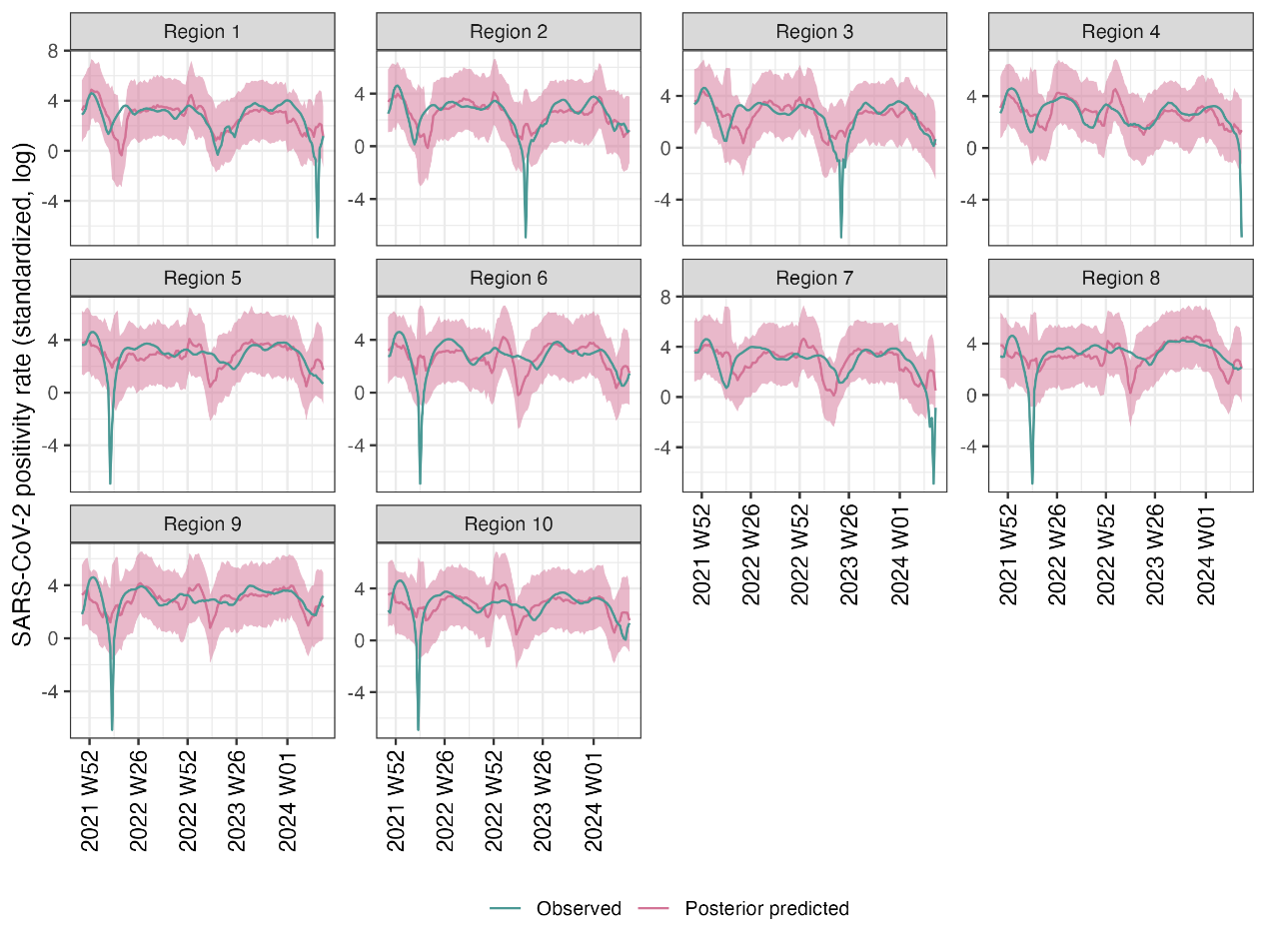
**

**Figure S3: Observed versus posterior predictive SARS-CoV-2 positivity rate (standardized, log) in United states, by HHS region.** Mean observed SARS-CoV-2 positivity rate (green curve) and corresponding posterior predictive mean (solid pink curve) and 95% prediction interval (shaded pink area) of SARS-CoV-2 positivity rate posterior predictive distributions from October 2021 to May 2024, simulated from the SARS-CoV-2 - IAV model (refitted 33 times, leaving out four weeks at a time).


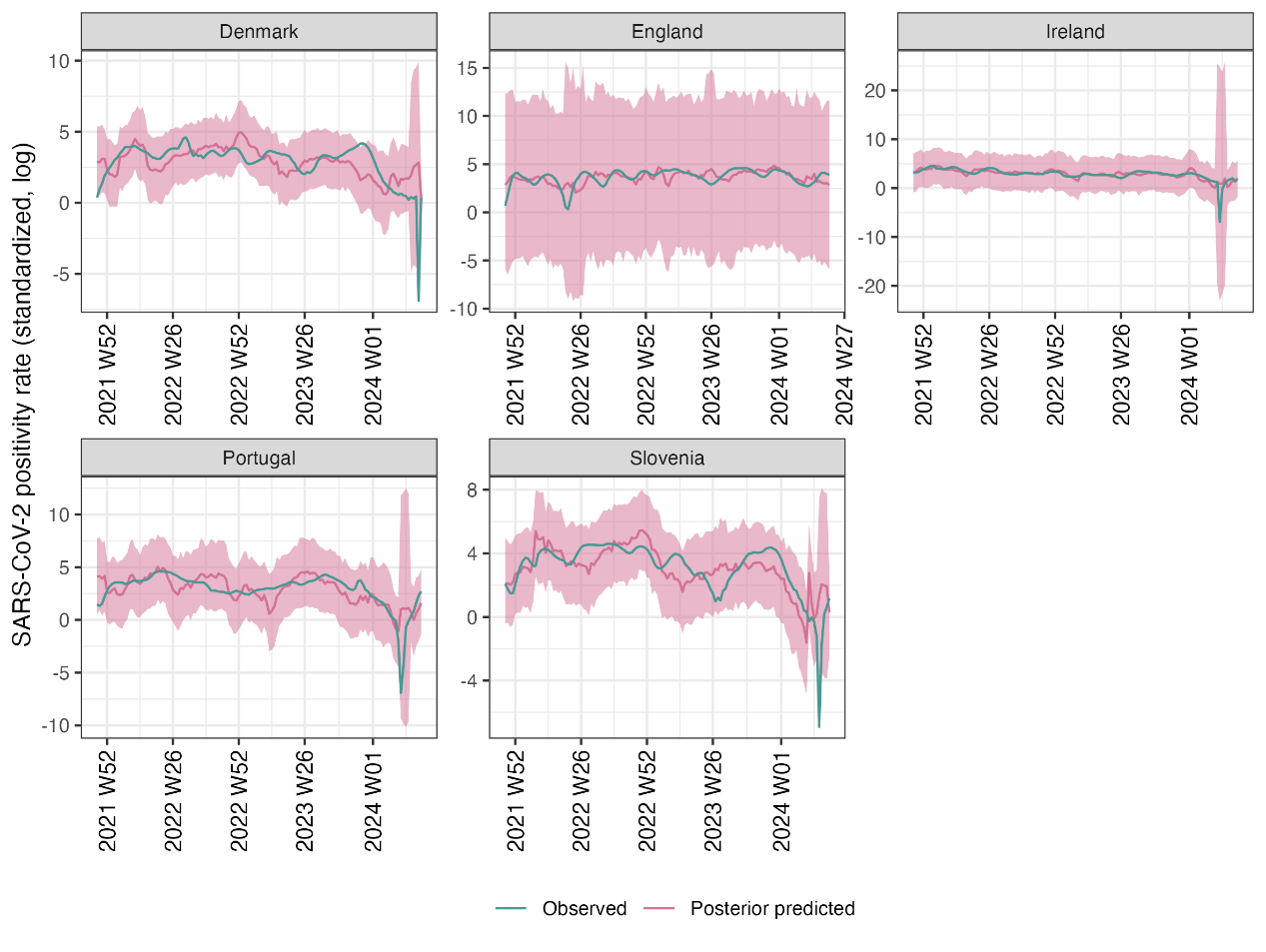


**Figure S4: Observed versus posterior predictive SARS-CoV-2 positivity rate (standardized, log) in five different locations and countries.** Mean observed SARS-CoV-2 positivity rate (green curve) and corresponding posterior predictive mean (solid pink curve) and 95% prediction interval (shaded pink area) of SARS-CoV-2 positivity rate posterior predictive distributions from October 2021 to May 2024, simulated from the SARS-CoV-2 - IAV model (refitted 33 times, leaving out four weeks at a time).

**
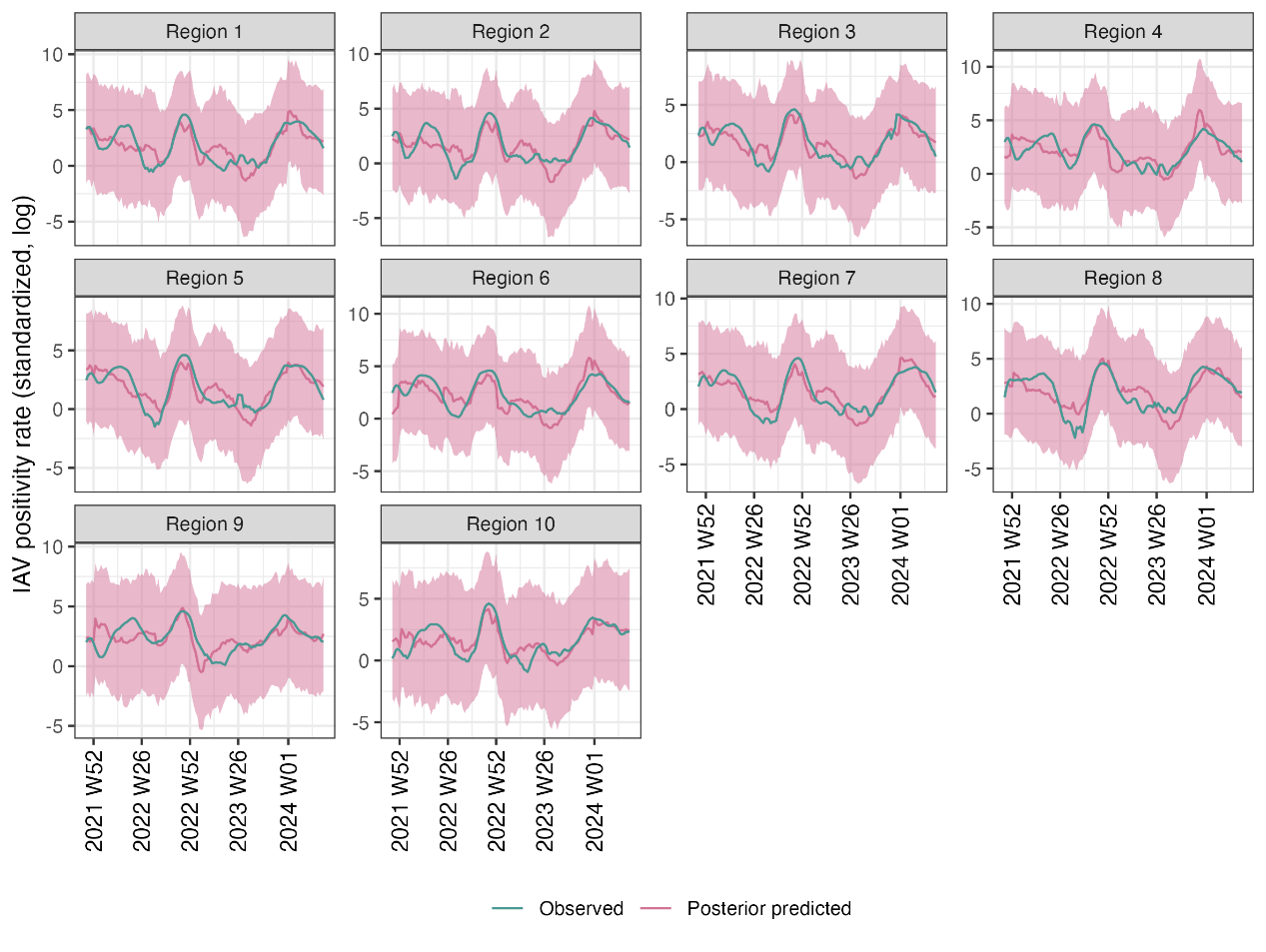
**

**Figure S5: Observed versus posterior predictive IAV positivity rate (standardized, log) in United states, by HHS region.** Mean observed IAV positivity rate (green curve) and corresponding posterior predictive mean (solid pink curve) and 95% prediction interval (shaded pink area) of IAV positivity rate posterior predictive distributions from October 2021 to May 2024, simulated from the IAV- RSV model (refitted 33 times, leaving out four weeks at a time).


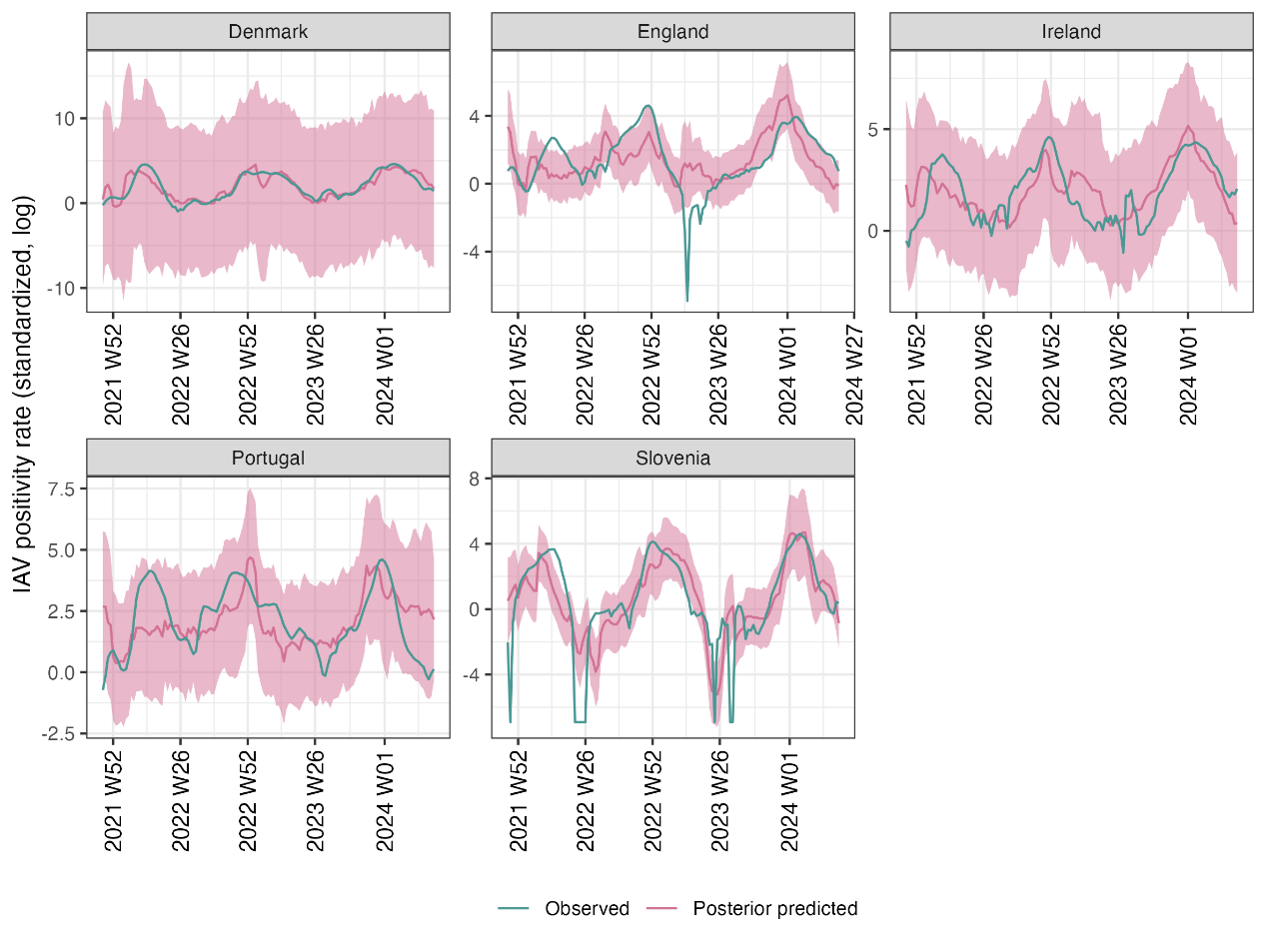


**Figure S6: Observed versus posterior predictive IAV positivity rate (standardized, log) in five different locations and countries.** Mean observed IAV positivity rate (green curve) and corresponding posterior predictive mean (solid pink curve) and 95% prediction interval (shaded pink area) of IAV positivity rate posterior predictive distributions from October 2021 to May 2024, simulated from the IAV- RSV model (refitted 33 times, leaving out four weeks at a time).

**
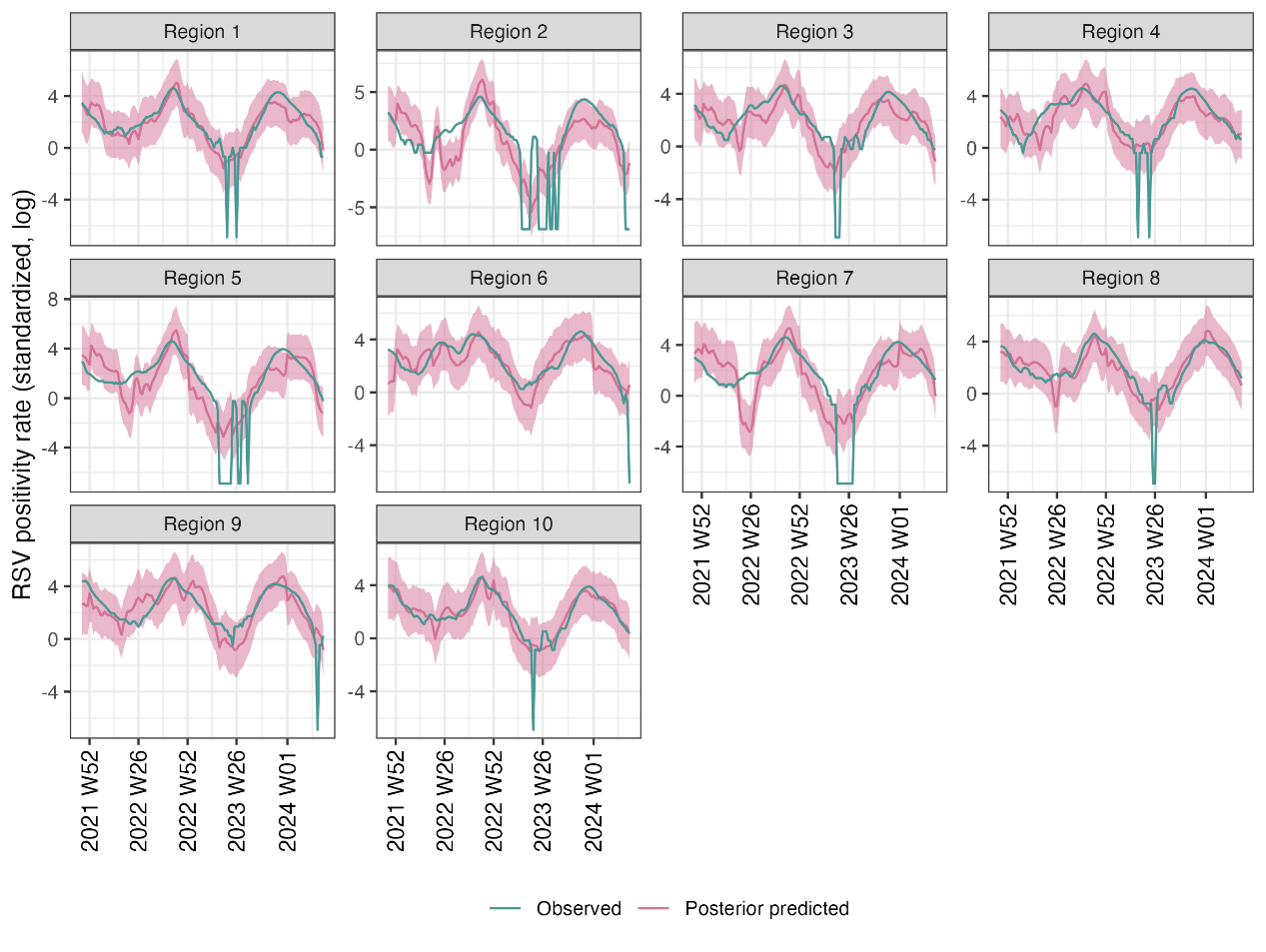
**

**Figure S7: Observed versus posterior predictive RSV positivity rate (standardized, log) in United states, by HHS region.** Mean observed RSV positivity rate (green curve) and corresponding posterior predictive mean (solid pink curve) and 95% prediction interval (shaded pink area) of RSV positivity rate posterior predictive distributions from October 2021 to May 2024, simulated from the RSV- IAV model (refitted 33 times, leaving out four weeks at a time).

**
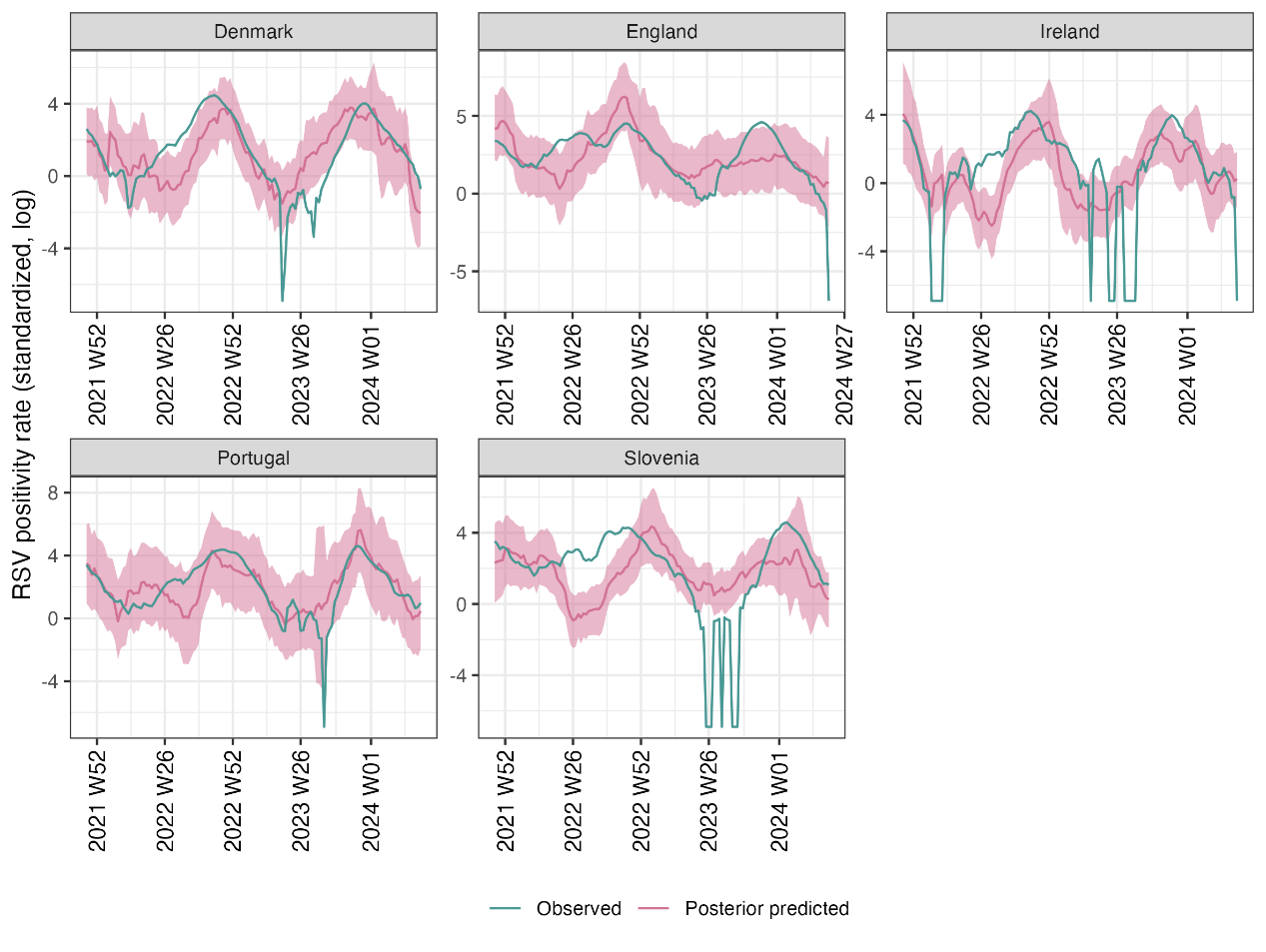
**

**Figure S8: Observed versus posterior predictive RSV positivity rate (standardized, log) in five different locations and countries.** Mean observed RSV positivity rate (green curve) and corresponding posterior predictive mean (solid pink curve) and 95% prediction interval (shaded pink area) of RSV positivity rate posterior predictive distributions from October 2021 to May 2024, simulated from the RSV- IAV model (refitted 33 times, leaving out four weeks at a time).

**
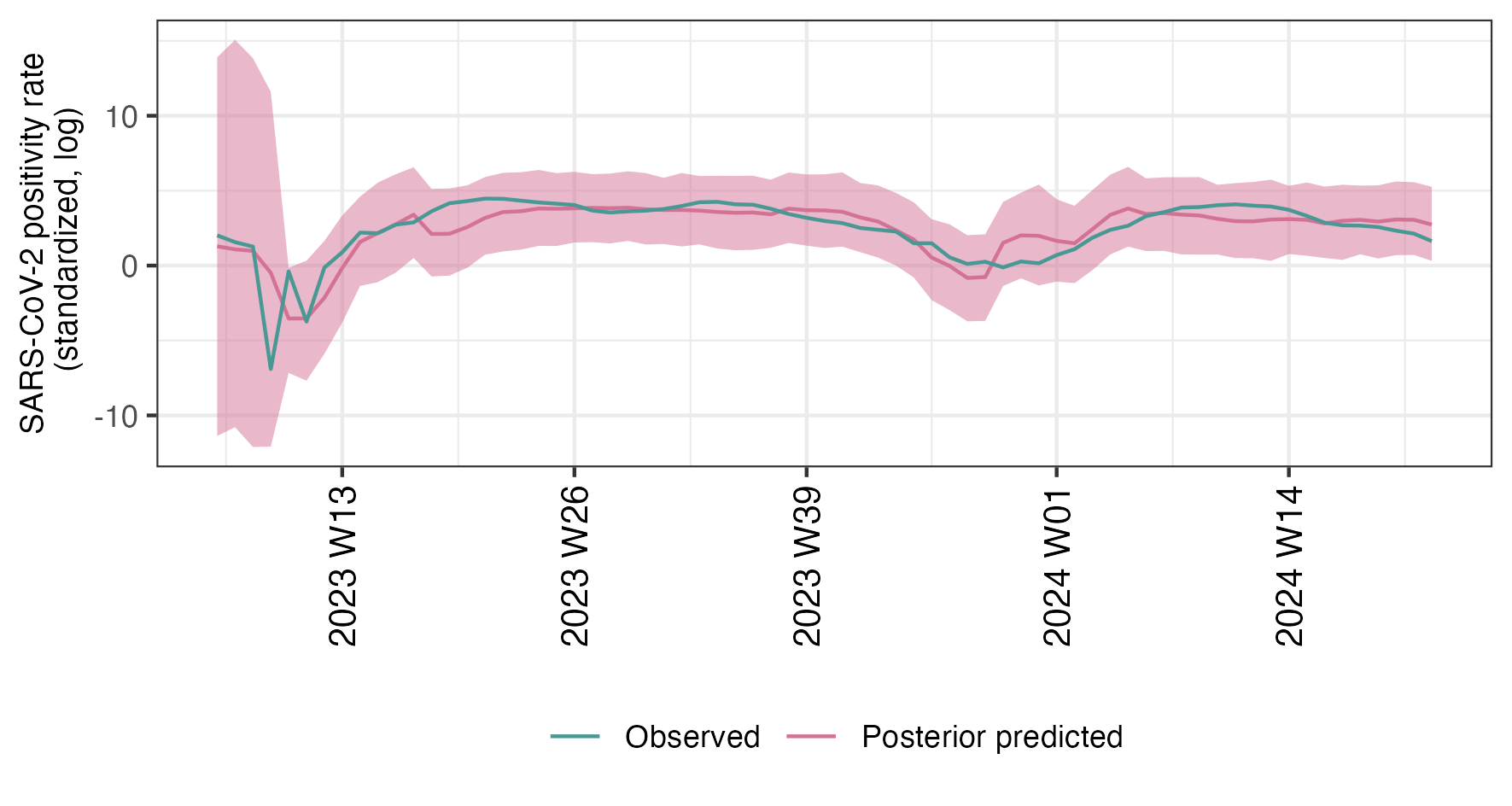
**

**Figure S9: Observed versus posterior predictive SARS-CoV-2 positivity rate (standardized, log) in Beijing.** Mean observed SARS-CoV-2 positivity rate (green curve) and corresponding posterior predictive mean (solid pink curve) and 95% prediction interval (shaded pink area) of SARS-CoV-2 positivity rate posterior predictive distributions from January 2023 to May 2024, simulated from the SARS-CoV-2 - IAV model (refitted 18 times, leaving out four weeks at a time).


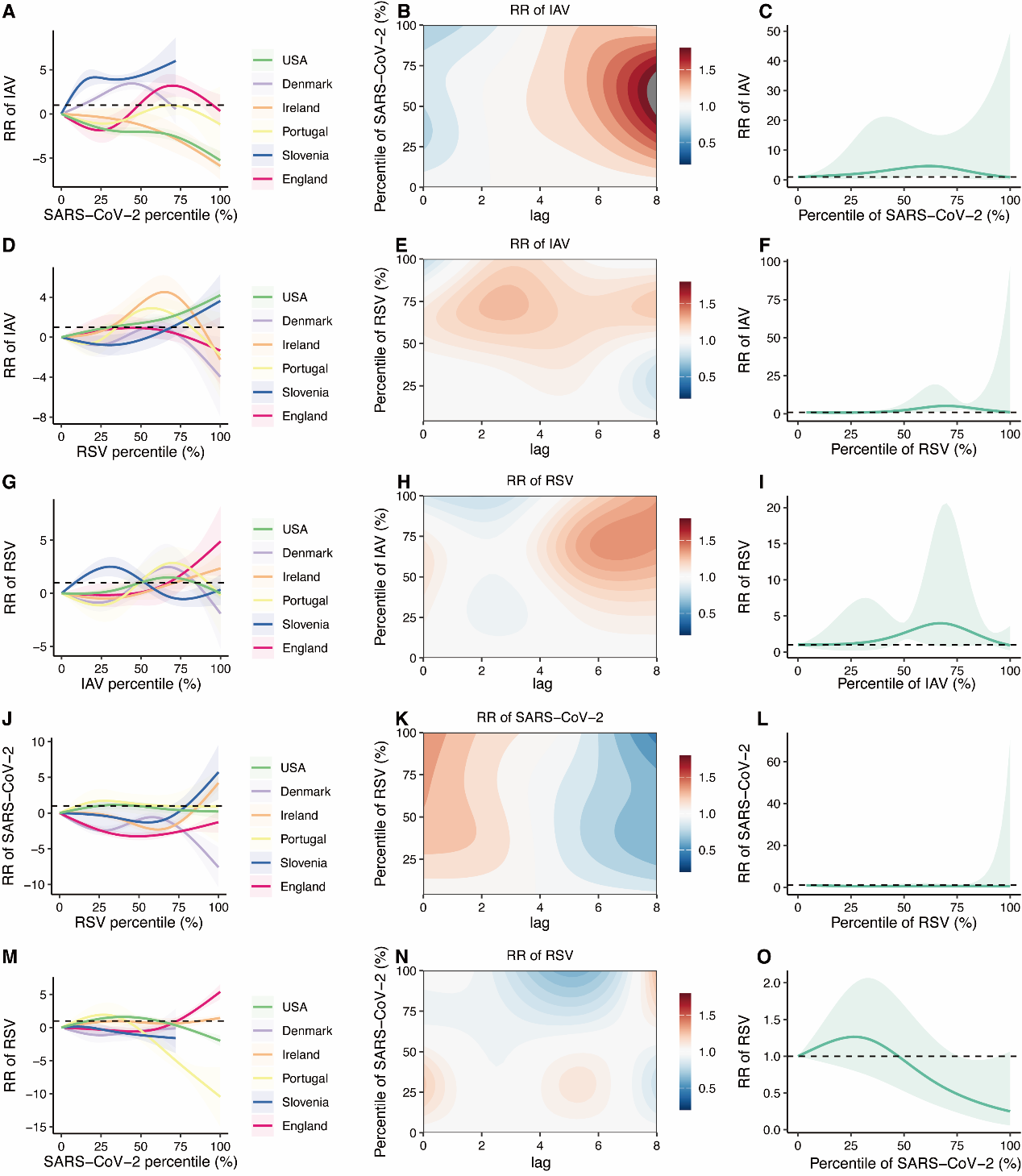


**Figure S10: Exposure-response association between viruses during epidemic seasons in 2021-2024.** Associations between percentile of SARS-CoV-2 positivity and subsequent IAV infection risk. (A) RR of IAV associated with different percentiles of SARS-CoV-2 positivity across regions, shaded areas representing 95% CrI. (B) Pooled contour plot of the lag–response association between percentile of SARS-CoV-2 positivity and IAV risk, relative to the minimum SARS-CoV-2 percentile. (C) Pooled exposure–response association between percentile of SARS-CoV-2 positivity and IAV risk, shaded areas representing 95% CrI. (D–F) Associations between percentile of RSV positivity and subsequent IAV infection risk. (G–I) Associations between percentile of IAV positivity and subsequent RSV infection risk. (J–L) Associations between percentile of RSV positivity and subsequent SARS-CoV-2 infection risk. (M–O) Associations between percentile of SARS-CoV-2 positivity and subsequent RSV infection risk. Estimates are based on n = 3510 biologically independent weekly observations included in the DLNM.


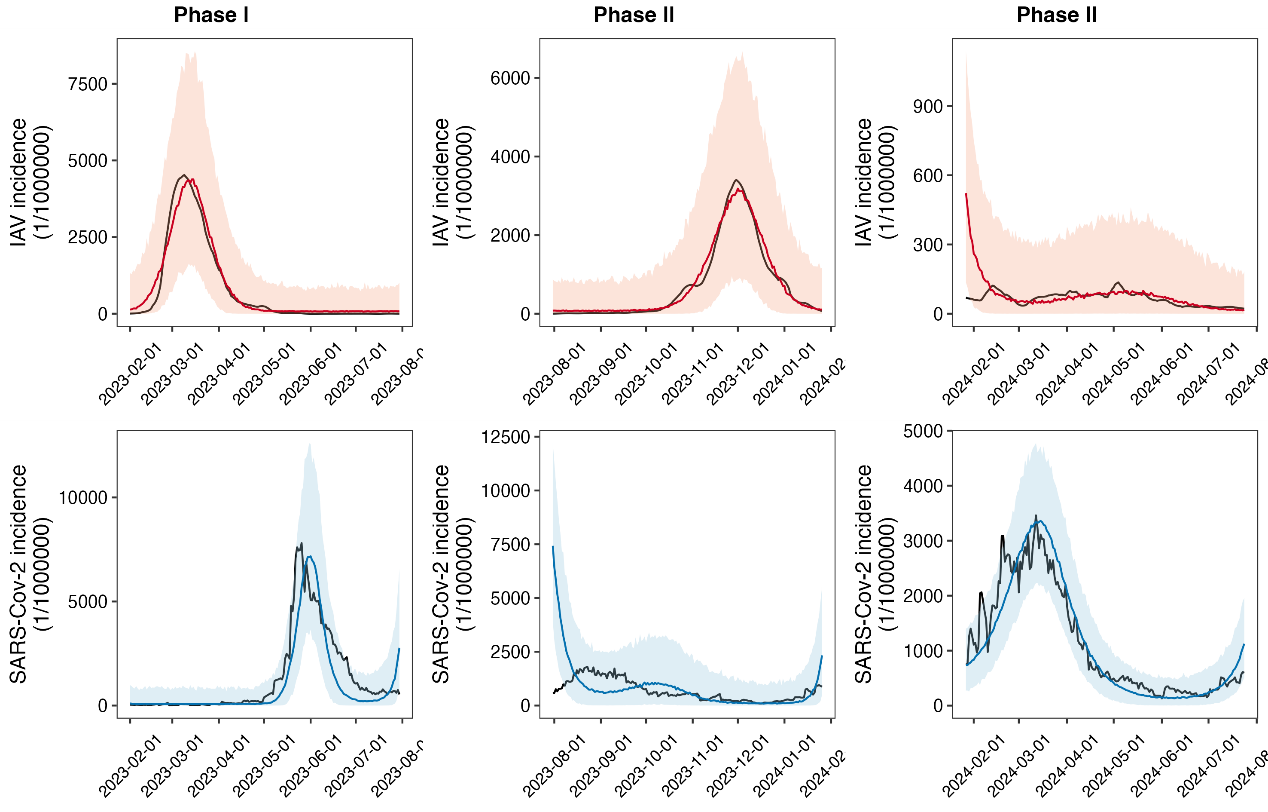


**Figure S11: Fitted transmission dynamic model with interactions between IAV and SARS-CoV-2 across three phases in Beijing.** (A) IAV. (B) SARS-CoV-2. Black lines represent the observed incidence, red lines represent the estimated incidence of IAV, blue lines represent the estimated incidence of SARS-CoV-2. Shaded areas representing 95% prediction interval, model was based on n = 541 biologically independent daily surveillance observations.

.

**
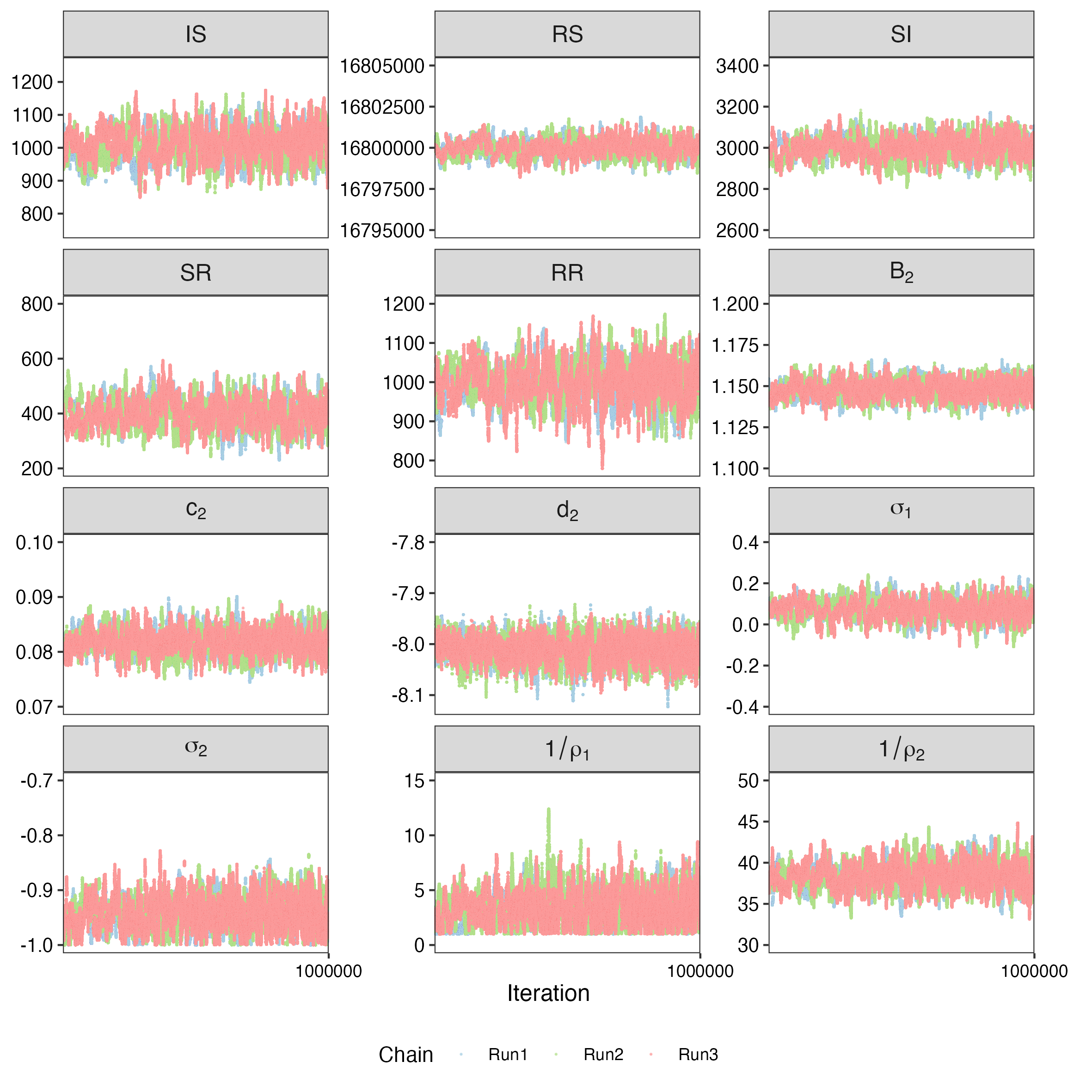
**

**Figure S12: Trace plots of parameter estimates from the epidemic model, obtained using Bayesian Markov Chain Monte Carlo (MCMC) methods. The three different colors represent results from three independent MCMC chains.**

**
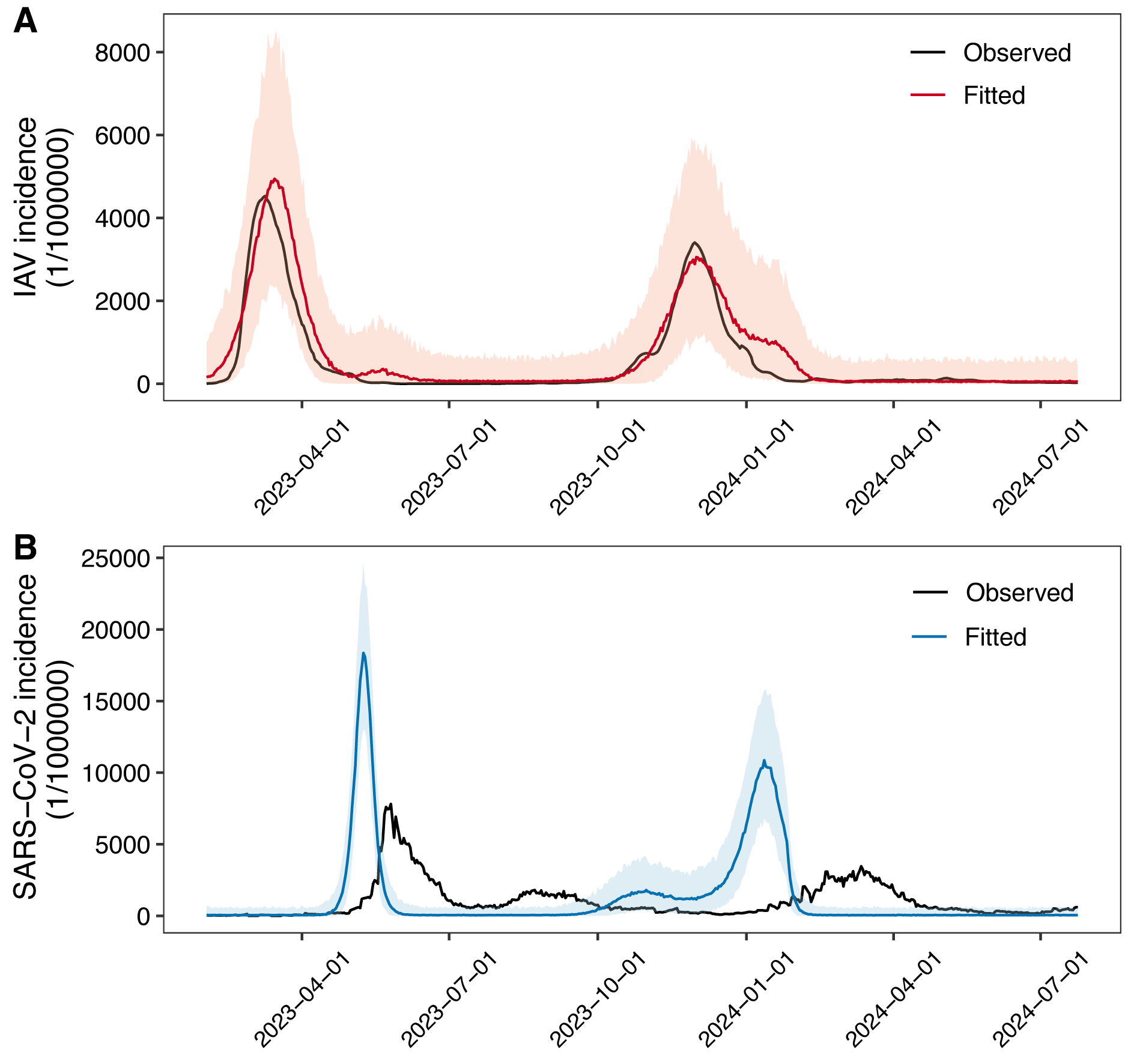
**

**Figure S13: Simulated epidemic trends of IAV and SARS-CoV-2 without interaction in Beijing.** (A) IAV. (B) SARS-CoV-2. Black lines represent the observed incidence, red lines represent the estimated incidence of IAV, blue lines represent the estimated incidence of SARS-CoV-2. Shaded areas representing 95% prediction interval, model was based on n = 541 biologically independent daily surveillance observations.

**Table S1: Model adequacy results for models of increasing complexity of modelling investigating the impact of IAV infection on SARS-CoV-2 risk in United states.** The deviance information criterion (DIC) and the cross-validated (CV) mean logarithmic score for models of increasing complexity. Lower scores indicate a better fitting model.

| **Model** | **SARS-CoV-2 percentile estimate** | **DIC** | **CV mean log score** |
| --- | --- | --- | --- |
| Baseline | $\alpha_{c, t}+ \mu_{c, t}+ \varphi_{c, t}$  Spatiotemporal random effects | 3793 | 1.484 |
| Temp | Base model + Temp | 3794 | 1.485 |
| RH | Base model + RH | 3793 | 1.484 |
| Popu density | Base model + Popu density | 3794 | 1.484 |
| Above65 | Base model + Above65 | 3794 | 1.484 |
| NPI | Base model + NPI | 3785 | 1.481 |
| Omicron | Base model + Omicron | 3795 | 1.484 |
| BA.2.75 | Base model + BA.2.75 | 3778 | 1.479 |
| BA.2.86 | Base model + BA.2.86 | 3795 | 1.484 |
| Immunity | Base model + Immunity | 3791 | 1.483 |
| Temp + Popu density + Above65 + NPI + Omicron + BA.2.75 + BA.2.86 + immunity | Base model + Temp + Popu density + Above65 + NPI + Omicron + BA.2.75 + BA.2.86 + immunity | 3760 | 1.475 |
| RH + Popu density + Above65 + NPI + Omicron + BA.2.75 + BA.2.86 + immunity | Base model + RH + Popu density + Above65 + NPI + Omicron + BA.2.75 + BA.2.86 + immunity | 3761 | 1.475 |
| RH + Popu density + Above65 + NPI + Omicron + BA.2.75 + BA.2.86 + immunity + IAV | Base model + RH + Popu density + Above65 + NPI + Omicron + BA.2.75 + BA.2.86 + immunity + IAV | 3681 | 1.443 |

Temp: mean temperature;

RH: relative humidty;

Popu density: Population density

Above65: Population ages 65 and above

NPI: Non-Pharmaceutical Interventions

Omicron: The proportion of Omicron variants (excluding XBB, BA.2.75 and BA.2.86) among COVID-19 variants.

BA.2.75: The proportion of BA.2.75 variant among COVID-19 variants.

BA.2.86: The proportion of BA.2.86 variant among COVID-19 variants.

IAV: Influenza A virus

**Table S2: Model adequacy results for models of increasing complexity of modelling investigating the impact of IAV infection on SARS-CoV-2 risk in Denmark.**

| **Model** | **SARS-CoV-2 percentile estimate** | **DIC** | **CV mean log score** |
| --- | --- | --- | --- |
| Baseline | $\alpha_{c, t}+ \mu_{c, t}+ \varphi_{c, t}$  Spatiotemporal random effects | 446 | 1.77 |
| Temp | Base model + Temp | 447 | 1.775 |
| RH | Base model + RH | 450 | 1.787 |
| NPI | Base model + NPI | 445 | 1.768 |
| Omicron | Base model + Omicron | 425 | 1.698 |
| BA.2.75 | Base model + BA.2.75 | 443 | 1.761 |
| BA.2.86 | Base model + BA.2.86 | 404 | 1.62 |
| Immunity | Base model + Immunity | 448 | 1.78 |
| Temp + NPI + Omicron + BA.2.75 + BA.2.86 + immunity | Base model + Temp + NPI + Omicron + BA.2.75 + BA.2.86 + immunity | 382 | 1.557 |
| RH + NPI + Omicron + BA.2.75 + BA.2.86 + immunity | Base model + RH + NPI + Omicron + BA.2.75 + BA.2.86 + immunity | 388 | 1.579 |
| RH + NPI + Omicron + BA.2.75 + BA.2.86 + immunity + IAV | Base model + RH + NPI + Omicron + BA.2.75 + BA.2.86 + immunity + IAV | 364 | 1.586 |

**Table S3: Model adequacy results for models of increasing complexity of modelling investigating the impact of IAV infection on SARS-CoV-2 risk in England.**

| **Model** | **SARS-CoV-2 percentile estimate** | **DIC** | **CV mean log score** |
| --- | --- | --- | --- |
| Baseline | $\alpha_{c, t}+ \mu_{c, t}+ \varphi_{c, t}$  Spatiotemporal random effects | 298 | 1.154 |
| Temp | Base model + Temp | 299 | 1.159 |
| RH | Base model + RH | 302 | 1.17 |
| NPI | Base model + NPI | 281 | 1.089 |
| Omicron | Base model + Omicron | 284 | 1.103 |
| BA.2.75 | Base model + BA.2.75 | 291 | 1.128 |
| BA.2.86 | Base model + BA.2.86 | 300 | 1.16 |
| Immunity | Base model + Immunity | 271 | 1.051 |
| Temp + NPI + Omicron + BA.2.75 + BA.2.86 + immunity | Base model + Temp + NPI + Omicron + BA.2.75 + BA.2.86 + immunity | 257 | 0.998 |
| RH + NPI + Omicron + BA.2.75 + BA.2.86 + immunity | Base model + RH + NPI + Omicron + BA.2.75 + BA.2.86 + immunity | 254 | 0.99 |
| RH + NPI + Omicron + BA.2.75 + BA.2.86 + immunity + IAV | Base model + RH + NPI + Omicron + BA.2.75 + BA.2.86 + immunity + IAV | 213 | 0.824 |

**Table S4: Model adequacy results for models of increasing complexity of modelling investigating the impact of IAV infection on SARS-CoV-2 risk in Ireland.**

| **Model** | **SARS-CoV-2 percentile estimate** | **DIC** | **CV mean log score** |
| --- | --- | --- | --- |
| Baseline | $\alpha_{c, t}+ \mu_{c, t}+ \varphi_{c, t}$  Spatiotemporal random effects | 403 | 1.647 |
| Temp | Base model + Temp | 404 | 1.651 |
| RH | Base model + RH | 407 | 1.665 |
| NPI | Base model + NPI | 393 | 1.619 |
| Omicron | Base model + Omicron | 373 | 1.555 |
| BA.2.75 | Base model + BA.2.75 | 404 | 1.65 |
| BA.2.86 | Base model + BA.2.86 | 357 | 1.499 |
| Immunity | Base model + Immunity | 406 | 1.658 |
| Temp + NPI + Omicron + BA.2.75 + BA.2.86 + immunity | Base model + Temp + NPI + Omicron + BA.2.75 + BA.2.86 + immunity | 327 | 1.46 |
| RH + NPI + Omicron + BA.2.75 + BA.2.86 + immunity | Base model + RH + NPI + Omicron + BA.2.75 + BA.2.86 + immunity | 335 | 1.465 |
| RH + NPI + Omicron + BA.2.75 + BA.2.86 + immunity + IAV | Base model + RH + NPI + Omicron + BA.2.75 + BA.2.86 + immunity + IAV | 204 | 1.463 |

**Table S5: Model adequacy results for models of increasing complexity of modelling investigating the impact of IAV infection on SARS-CoV-2 risk in Portugal.**

| **Model** | **SARS-CoV-2 percentile estimate** | **DIC** | **CV mean log score** |
| --- | --- | --- | --- |
| Baseline | $\alpha_{c, t}+ \mu_{c, t}+ \varphi_{c, t}$  Spatiotemporal random effects | 461 | 1.816 |
| Temp | Base model + Temp | 465 | 1.843 |
| RH | Base model + RH | 464 | 1.828 |
| NPI | Base model + NPI | 459 | 1.811 |
| Omicron | Base model + Omicron | 441 | 1.745 |
| BA.2.75 | Base model + BA.2.75 | 460 | 1.815 |
| BA.2.86 | Base model + BA.2.86 | 412 | 1.638 |
| Immunity | Base model + Immunity | 444 | 1.757 |
| Temp + NPI + Omicron + BA.2.75 + BA.2.86 + immunity | Base model + Temp + NPI + Omicron + BA.2.75 + BA.2.86 + immunity | 392 | 1.574 |
| RH + NPI + Omicron + BA.2.75 + BA.2.86 + immunity | Base model + RH + NPI + Omicron + BA.2.75 + BA.2.86 + immunity | 392 | 1.563 |
| RH + NPI + Omicron + BA.2.75 + BA.2.86 + immunity + IAV | Base model + RH + NPI + Omicron + BA.2.75 + BA.2.86 + immunity + IAV | 341 | 1.401 |

**Table S6: Model adequacy results for models of increasing complexity of modelling investigating the impact of IAV infection on SARS-CoV-2 risk in Slovenia.**

| **Model** | **SARS-CoV-2 percentile estimate** | **DIC** | **CV mean log score** |
| --- | --- | --- | --- |
| Baseline | $\alpha_{c, t}+ \mu_{c, t}+ \varphi_{c, t}$  Spatiotemporal random effects | 479 | 1.878 |
| Temp | Base model + Temp | 480 | 1.883 |
| RH | Base model + RH | 486 | 1.904 |
| NPI | Base model + NPI | 479 | 1.878 |
| Omicron | Base model + Omicron | 429 | 1.7 |
| BA.2.75 | Base model + BA.2.75 | 478 | 1.873 |
| BA.2.86 | Base model + BA.2.86 | 416 | 1.638 |
| Immunity | Base model + Immunity | 478 | 1.873 |
| Temp + NPI + Omicron + BA.2.75 + BA.2.86 + immunity | Base model + Temp + NPI + Omicron + BA.2.75 + BA.2.86 + immunity | 373 | 1.495 |
| RH + NPI + Omicron + BA.2.75 + BA.2.86 + immunity | Base model + RH + NPI + Omicron + BA.2.75 + BA.2.86 + immunity | 378 | 1.514 |
| RH + NPI + Omicron + BA.2.75 + BA.2.86 + immunity + IAV | Base model + RH + NPI + Omicron + BA.2.75 + BA.2.86 + immunity + IAV | 354 | 1.466 |

**Table S7: Model adequacy results for models of increasing complexity of modelling investigating virus interactions the impact of RSV infection on IAV risk in United states.** The deviance information criterion (DIC) and the cross-validated (CV) mean logarithmic score for models of increasing complexity. Lower scores indicate a better fitting model.

| **Model** | **IAV percentile estimate** | **DIC** | **CV mean log score** |
| --- | --- | --- | --- |
| Baseline | $\alpha_{c, t}+ \mu_{c, t}+ \varphi_{c, t}$  Spatiotemporal random effects | 3203 | 1.244 |
| Temp | Base model + Temp | 3205 | 1.245 |
| RH | Base model + RH | 3202 | 1.244 |
| Popu density | Base model + Popu density | 3203 | 1.244 |
| Above65 | Base model + Above65 | 3202 | 1.244 |
| NPI | Base model + NPI | 3135 | 1.219 |
| Immunity | Base model + Immunity | 3187 | 1.238 |
| Temp + Popu density + Above65 + NPI + immunity | Base model + Temp + Popu density + Above65 + NPI + immunity | 3103 | 1.207 |
| RH + Popu density + Above65 + NPI + immunity | Base model + RH + Popu density + Above65 + NPI + immunity | 3098 | 1.206 |
| RH + Popu density + Above65 + NPI + immunity +RSV | Base model + RH + Popu density + Above65 + NPI + immunity +RSV | 2744 | 1.067 |

**Table S8: Model adequacy results for models of increasing complexity of modelling investigating virus interactions the impact of RSV infection on IAV risk in Denmark.**

| **Model** | **IAV percentile estimate** | **DIC** | **CV mean log score** |
| --- | --- | --- | --- |
| Baseline | $\alpha_{c, t}+ \mu_{c, t}+ \varphi_{c, t}$  Spatiotemporal random effects | 379 | 1.47 |
| Temp | Base model + Temp | 376 | 1.459 |
| RH | Base model + RH | 381 | 1.478 |
| NPI | Base model + NPI | 273 | 1.063 |
| Immunity | Base model + Immunity | 341 | 1.325 |
| Temp + NPI + immunity | Base model + Temp + NPI + immunity | 262 | 1.021 |
| RH + NPI + immunity | Base model + RH + NPI + immunity | 264 | 1.03 |
| RH + NPI + immunity +RSV | Base model + RH + NPI + immunity +RSV | 214 | 0.838 |

**Table S9: Model adequacy results for models of increasing complexity of modelling investigating virus interactions the impact of RSV infection on IAV risk in England.**

| **Model** | **IAV percentile estimate** | **DIC** | **CV mean log score** |
| --- | --- | --- | --- |
| Baseline | $\alpha_{c, t}+ \mu_{c, t}+ \varphi_{c, t}$  Spatiotemporal random effects | 484 | 1.872 |
| Temp | Base model + Temp | 486 | 1.877 |
| RH | Base model + RH | 486 | 1.877 |
| NPI | Base model + NPI | 472 | 1.827 |
| Immunity | Base model + Immunity | 486 | 1.88 |
| Temp + NPI + immunity | Base model + Temp + NPI + immunity | 461 | 1.785 |
| RH + NPI + immunity | Base model + RH + NPI + immunity | 463 | 1.791 |
| RH + NPI + immunity +RSV | Base model + RH + NPI + immunity +RSV | 439 | 1.697 |

**Table S10: Model adequacy results for models of increasing complexity of modelling investigating virus interactions the impact of RSV infection on IAV risk in Ireland.**

| **Model** | **IAV percentile estimate** | **DIC** | **CV mean log score** |
| --- | --- | --- | --- |
| Baseline | $\alpha_{c, t}+ \mu_{c, t}+ \varphi_{c, t}$  Spatiotemporal random effects | 415 | 1.61 |
| Temp | Base model + Temp | 412 | 1.597 |
| RH | Base model + RH | 416 | 1.613 |
| NPI | Base model + NPI | 365 | 1.415 |
| Immunity | Base model + Immunity | 417 | 1.617 |
| Temp + NPI + immunity | Base model + Temp + NPI + immunity | 341 | 1.323 |
| RH + NPI + immunity | Base model + RH + NPI + immunity | 344 | 1.335 |
| RH + NPI + immunity +RSV | Base model + RH + NPI + immunity +RSV | 328 | 1.270 |

**Table S11: Model adequacy results for models of increasing complexity of modelling investigating virus interactions the impact of RSV infection on IAV risk in Portugal.**

| **Model** | **IAV percentile estimate** | **DIC** | **CV mean log score** |
| --- | --- | --- | --- |
| Baseline | $\alpha_{c, t}+ \mu_{c, t}+ \varphi_{c, t}$  Spatiotemporal random effects | 428 | 1.658 |
| Temp | Base model + Temp | 428 | 1.659 |
| RH | Base model + RH | 423 | 1.64 |
| NPI | Base model + NPI | 385 | 1.493 |
| Immunity | Base model + Immunity | 429 | 1.661 |
| Temp + NPI + immunity | Base model + Temp + NPI + immunity | 378 | 1.466 |
| RH + NPI + immunity | Base model + RH + NPI + immunity | 377 | 1.46 |
| RH + NPI + immunity +RSV | Base model + RH + NPI + immunity +RSV | 358 | 1.381 |

**Table S12: Model adequacy results for models of increasing complexity of modelling investigating virus interactions the impact of RSV infection on IAV risk in Slovenia.**

| **Model** | **IAV percentile estimate** | **DIC** | **CV mean log score** |
| --- | --- | --- | --- |
| Baseline | $\alpha_{c, t}+ \mu_{c, t}+ \varphi_{c, t}$  Spatiotemporal random effects | 511 | 1.999 |
| Temp | Base model + Temp | 513 | 2.006 |
| RH | Base model + RH | 511 | 1.999 |
| NPI | Base model + NPI | 481 | 1.89 |
| Immunity | Base model + Immunity | 510 | 1.997 |
| Temp + NPI + immunity | Base model + Temp + NPI + immunity | 480 | 1.886 |
| RH + NPI + immunity | Base model + RH + NPI + immunity | 480 | 1.886 |
| RH + NPI + immunity +RSV | Base model + RH + NPI + immunity +RSV | 481 | 1.885 |

**Table S13: Model adequacy results for models of increasing complexity of modelling investigating virus interactions the impact of IAV infection on RSV risk in United states.** The deviance information criterion (DIC) and the cross-validated (CV) mean logarithmic score for models of increasing complexity. Lower scores indicate a better fitting model.

| **Model** | **RSV percentile estimate** | **DIC** | **CV mean log score** |
| --- | --- | --- | --- |
| Baseline | $\alpha_{c, t}+ \mu_{c, t}+ \varphi_{c, t}$  Spatiotemporal random effects | 4535 | 1.763 |
| Temp | Base model + Temp | 4525 | 1.76 |
| RH | Base model + RH | 4536 | 1.764 |
| Popu density | Base model + Popu density | 4535 | 1.763 |
| Above65 | Base model + Above65 | 4535 | 1.763 |
| NPI | Base model + NPI | 4435 | 1.725 |
| Immunity | Base model + Immunity | 4501 | 1.75 |
| Temp + Popu density + Above65 + NPI + immunity | Base model + Temp + Popu density + Above65 + NPI + immunity | 4331 | 1.686 |
| RH + Popu density + Above65 + NPI + immunity | Base model + RH + Popu density + Above65 + NPI + immunity | 4335 | 1.687 |
| RH + Popu density + Above65 + NPI + immunity + IAV | Base model + RH + Popu density + Above65 + NPI + immunity + IAV | 10012 | 3.92 |

**Table S14: Model adequacy results for models of increasing complexity of modelling investigating virus interactions the impact of IAV infection on RSV risk in Denmark.**

| **Model** | **RSV percentile estimate** | **DIC** | **CV mean log score** |
| --- | --- | --- | --- |
| Baseline | $\alpha_{c, t}+ \mu_{c, t}+ \varphi_{c, t}$  Spatiotemporal random effects | 466 | 1.812 |
| Temp | Base model + Temp | 467 | 1.816 |
| RH | Base model + RH | 458 | 1.784 |
| NPI | Base model + NPI | 455 | 1.77 |
| Immunity | Base model + Immunity | 461 | 1.794 |
| Temp + NPI + immunity | Base model + Temp + NPI + immunity | 457 | 1.778 |
| RH + NPI + immunity | Base model + RH + NPI + immunity | 447 | 1.738 |
| RH + NPI + immunity + IAV | Base model + RH + NPI + immunity + IAV | 435 | 1.680 |

**Table S15: Model adequacy results for models of increasing complexity of modelling investigating virus interactions the impact of IAV infection on RSV risk in England.**

| **Model** | **RSV percentile estimate** | **DIC** | **CV mean log score** |
| --- | --- | --- | --- |
| Baseline | $\alpha_{c, t}+ \mu_{c, t}+ \varphi_{c, t}$  Spatiotemporal random effects | 434 | 1.702 |
| Temp | Base model + Temp | 436 | 1.707 |
| RH | Base model + RH | 438 | 1.716 |
| NPI | Base model + NPI | 435 | 1.704 |
| Immunity | Base model + Immunity | 415 | 1.627 |
| Temp + NPI + immunity | Base model + Temp + NPI + immunity | 418 | 1.635 |
| RH + NPI + immunity | Base model + RH + NPI + immunity | 418 | 1.636 |
| RH + NPI + immunity + IAV | Base model + RH + NPI + immunity + IAV | 408 | 1.593 |

**Table S16: Model adequacy results for models of increasing complexity of modelling investigating virus interactions the impact of IAV infection on RSV risk in Ireland.**

| **Model** | **RSV percentile estimate** | **DIC** | **CV mean log score** |
| --- | --- | --- | --- |
| Baseline | $\alpha_{c, t}+ \mu_{c, t}+ \varphi_{c, t}$  Spatiotemporal random effects | 608 | 2.362 |
| Temp | Base model + Temp | 608 | 2.364 |
| RH | Base model + RH | 608 | 2.363 |
| NPI | Base model + NPI | 610 | 2.367 |
| Immunity | Base model + Immunity | 609 | 2.366 |
| Temp + NPI + immunity | Base model + Temp + NPI + immunity | 612 | 2.375 |
| RH + NPI + immunity | Base model + RH + NPI + immunity | 612 | 2.374 |
| RH + NPI + immunity + IAV | Base model + RH + NPI + immunity + IAV | 589 | 2.284 |

**Table S17: Model adequacy results for models of increasing complexity of modelling investigating virus interactions the impact of IAV infection on RSV risk in Portugal.**

| **Model** | **RSV percentile estimate** | **DIC** | **CV mean log score** |
| --- | --- | --- | --- |
| Baseline | $\alpha_{c, t}+ \mu_{c, t}+ \varphi_{c, t}$  Spatiotemporal random effects | 422 | 1.672 |
| Temp | Base model + Temp | 423 | 1.675 |
| RH | Base model + RH | 416 | 1.649 |
| NPI | Base model + NPI | 413 | 1.647 |
| Immunity | Base model + Immunity | 421 | 1.667 |
| Temp + NPI + immunity | Base model + Temp + NPI + immunity | 417 | 1.656 |
| RH + NPI + immunity | Base model + RH + NPI + immunity | 410 | 1.632 |
| RH + NPI + immunity + IAV | Base model + RH + NPI + immunity + IAV | 371 | 1.490 |

**Table S18: Model adequacy results for models of increasing complexity of modelling investigating virus interactions the impact of IAV infection on RSV risk in Slovenia.**

| **Model** | **RSV percentile estimate** | **DIC** | **CV mean log score** |
| --- | --- | --- | --- |
| Baseline | $\alpha_{c, t}+ \mu_{c, t}+ \varphi_{c, t}$  Spatiotemporal random effects | 511 | 1.999 |
| Temp | Base model + Temp | 513 | 2.006 |
| RH | Base model + RH | 511 | 1.999 |
| NPI | Base model + NPI | 481 | 1.89 |
| Immunity | Base model + Immunity | 510 | 1.997 |
| Temp + NPI + immunity | Base model + Temp + NPI + immunity | 480 | 1.886 |
| RH + NPI + immunity | Base model + RH + NPI + immunity | 480 | 1.886 |
| RH + NPI + immunity + IAV | Base model + RH + NPI + immunity + IAV | 481 | 1.885 |

**Table S19: Multivariate meta-regression models for meta-predictors of the effect of IAV infection on SARS-CoV-2 risk.**

| **Model** | **AIC** | **Q** | **df** | **p** | **I^2^** |
| --- | --- | --- | --- | --- | --- |
| Baseline | 266.36 | 604.52 | 60.00 | <2.2 × 10⁻¹⁶ | 90.07 |
| Popu density | 401.38 | 393.90 | 48.00 | <2.2 × 10⁻¹⁶ | 87.81 |
| SDI | 214.68 | 509.34 | 48.00 | <2.2 × 10⁻¹⁶ | 90.58 |
| Above65 | 317.53 | 430.14 | 48.00 | <2.2 × 10⁻¹⁶ | 88.84 |
| Popu density + SDI + Above65 | 398.96 | 192.02 | 24.00 | <2.2 × 10⁻¹⁶ | 87.50 |

Popu density: Population density

SDI: Socio-demographic index

Above65: Population ages 65 and above

**References**

1. The Center for Disease Control and Prevention. The National Respiratory and Enteric Virus Surveillance System (NREVSS). <https://www.cdc.gov/surveillance/nrevss/index.html>.

2. Zhao, H. *et al.* A new laboratory-based surveillance system (Respiratory DataMart System) for influenza and other respiratory viruses in England: results and experience from 2009 to 2012. *Eurosurveillance* **19**, (2014).

3. European Centre for Disease Prevention and Control. The European Surveillance System (TESSy). <https://www.ecdc.europa.eu/en/publications-data/european-surveillance-system-tessy>.

4. Li, M. *et al.* Characterising the changes in RSV epidemiology in Beijing, China during 2015–2023: results from a prospective, multi-centre, hospital-based surveillance and serology study. *Lancet Reg. Heal. - West. Pac.* **45**, 101050 (2024).

5. Nickbakhsh, S. *et al.* Virus–virus interactions impact the population dynamics of influenza and the common cold. *Proc. Natl. Acad. Sci.* **116**, 27142–27150 (2019).

6. Manathunga, S. S., Abeyagunawardena, I. A. & Dharmaratne, S. D. A comparison of transmissibility of SARS-CoV-2 variants of concern. *Virol. J.* **20**, 59 (2023).

7. Wong, A. *et al.* The interactions of SARS-CoV-2 with cocirculating pathogens: Epidemiological implications and current knowledge gaps. *PLOS Pathog.* **19**, e1011167 (2023).

8. U.S. centers for disease control and prevention. About Reinfection. <https://www.cdc.gov/covid/about/reinfection.html#:~:text=Once%20you%20have%20had%20COVID>,response%20or%20none%20at%20all.

9. Waterlow, N. R., Flasche, S., Minter, A. & Eggo, R. M. Competition between RSV and influenza: Limits of modelling inference from surveillance data. *Epidemics* **35**, 100460 (2021).

10. SUKENO, N. *et al.* Anti-Nucleoprotein Antibody Response in Influenza A Infection. *Tohoku J. Exp. Med.* **128**, 241 (2008).

11. Moore, H. C., Jacoby, P., Hogan, A. B., Blyth, C. C. & Mercer, G. N. Modelling the Seasonal Epidemics of Respiratory Syncytial Virus in Young Children. *PLoS ONE* **9**, e100422 (2014).
